# Structural Integration as an Adjunct to Outpatient Rehabilitation for Chronic Nonspecific Low Back Pain: A Randomized Pilot Clinical Trial

**DOI:** 10.1155/2015/813418

**Published:** 2015-04-07

**Authors:** Eric E. Jacobson, Alec L. Meleger, Paolo Bonato, Peter M. Wayne, Helene M. Langevin, Ted J. Kaptchuk, Roger B. Davis

**Affiliations:** ^1^Department of Global Health & Social Medicine, Harvard Medical School, 641 Huntington Avenue, Boston, MA 02115, USA; ^2^Department of Physical Therapy & Rehabilitation, Harvard Medical School, Spaulding Rehabilitation Hospital, 300 First Avenue, Charleston, MA 02129, USA; ^3^Spine Center, Newton-Wellesley Hospital, 159 Wells Avenue, Newton, MA 02459, USA; ^4^Motion Analysis Laboratory, Department of Physical Therapy & Rehabilitation, Spaulding Rehabilitation Hospital, 300 First Avenue, Charleston, MA 02129, USA; ^5^Harvard-MIT Division of Health Sciences and Technology, 45 Carleton Street, Cambridge, MA 02142, USA; ^6^Osher Center for Integrative Medicine, Division of Preventive Medicine, Harvard Medical School and Brigham and Women's Hospital, 900 Commonwealth Avenue, Boston, MA 02215, USA; ^7^Department of Neurological Sciences, College of Medicine, University of Vermont, 89 Beaumont Avenue, Burlington, VT 05401, USA; ^8^Division of General Medicine & Primary Care, Harvard Medical School and Beth Israel Deaconess Medical Center, 330 Brookline Avenue, Boston, MA 02215, USA

## Abstract

Structural Integration (SI) is an alternative method of manipulation and movement education. To obtain preliminary data on feasibility, effectiveness, and adverse events (AE), 46 outpatients from Boston area with chronic nonspecific low back pain (CNSLBP) were randomized to parallel treatment groups of SI plus outpatient rehabilitation (OR) *versus* OR alone. Feasibility data were acceptable except for low compliance with OR and lengthy recruitment time. Intent-to-treat data on effectiveness were analyzed by Wilcoxon rank sum, *n* = 23 per group. Median reductions in VAS Pain, the primary outcome, of −26 mm in SI + OR *versus* 0 in OR alone were not significantly different (*P* = 0.075). Median reductions in RMDQ, the secondary outcome, of −2 points in SI + OR *versus* 0 in OR alone were significantly different (*P* = 0.007). Neither the proportions of participants with nor the seriousness of AE were significantly different. SI as an adjunct to OR for CNSLBP is not likely to provide additional reductions in pain but is likely to augment short term improvements in disability with a low additional burden of AE. A more definitive trial is feasible, but OR compliance and recruitment might be challenging. This trial is registered with ClinicalTrials.gov (NCT01322399).

## 1. Introduction

Chronic low back pain is among the most burdensome of health problems in prevalence and cost of care [1]. It is the leading cause of years lived with disability worldwide and the most frequent cause of functional loss in high-income countries [2, 3]. Much of the economic burden is expended on costly surgical and rehabilitative services. Up to one-third of acute low back pain cases may become chronic and lead to disability [4]. In a majority of chronic cases (estimated at 85–95%) a definitive diagnosis, that is, infection, neoplasm, osteoporosis, arthritis, fracture, radiculopathy, or inflammatory rheumatic processes, is ruled out, and these are designated as chronic “uncomplicated,” “mechanical,” or “nonspecific” low back pain (CNSLBP) [5]. There is no consensus on the optimal approach to the treatment of CNSLBP. Management typically includes some combination of analgesic or anti-inflammatory medication, directed therapeutic exercise, manipulation, cognitive-behavioral therapy, and patient education [6]. Systematic reviews have generally concluded that the benefits of these approaches are limited and mostly short-lived [7–11]. A large survey in the United States found that 54% of patients with low back or neck pain used complementary therapies and that approximately one-third of all visits to alternative care practices were for back or neck pain [12]. Low back pain has been reported to be the primary complaint in 40% of all visits to chiropractors, 20% to massage therapists, and 15% to acupuncturists [13].

Structural Integration (SI) is an alternative manual therapy that is increasingly available and sometimes resorted to for the treatment of chronic musculoskeletal pain and disability. Developed by the biochemist Ida Rolf outside of orthodox medical science, it has been propagated as an alternative therapy since the mid-1950s. A few preliminary studies of low quality with small samples suggest effectiveness for musculoskeletal pain, but aside from a single case report, no clinical studies of SI for CNSLBP have been published to date [14–16]. The musculoskeletal pain studies and preliminary evidence regarding a number of hypothesized therapeutic mechanisms have been reviewed elsewhere [17].

The experience of SI treatment sometimes involves notable discomfort which has led to a reputation of being excessively painful and even to concerns as to its safety [18]. This has been a barrier to a more widespread adoption by conventional clinical services, although SI was successfully incorporated into at least one [15, 19]. Despite these concerns, published data on adverse events (AE) associated with SI are limited to a single case and a small prospective case series [20, 21].

This study was designed to collect preliminary data on the feasibility, effectiveness, and AE associated with SI as an adjunct to outpatient rehabilitation (OR)* versus* OR alone for CNSLBP. The outcomes will inform the design of a more adequately powered clinical trial. We hypothesized that we could recruit and retain qualified participants who would comply with treatment regimens and data collection, that a course of SI + OR would improve low back related pain and disability significantly more than OR alone, and that SI could be delivered with acceptable levels of AE.

## 2. Methods

### 2.1. Design

Following baseline data collection, participants were randomized 1 : 1, and open label to parallel treatment groups. Follow-up data were collected at 20 weeks after baseline.

### 2.2. Setting, Recruitment, and Enrollment

The study was conducted at the Motion Analysis Laboratory (MAL), Spaulding Rehabilitation Hospital, Partners HealthCare, LLC, Boston, and was approved by the Spaulding institutional review board (IRB) (2010-P-00004). An independent monitor and a data-monitoring group provided additional oversight. The study was registered with ClinicalTrials.gov (NCT01322399) prior to beginning recruitment.

We included men and women aged 18–65 residing in the greater Boston area, with CNSLBP of ≥6 months duration not attributed to infection, neoplasm, severe radiculopathy (assessed by frequent severe pain radiating down a leg), fracture, or inflammatory rheumatic process, with a patient-rated bothersomeness of pain on average over the preceding 6 months ≥3 on an 11-point ordinal scale (0 = none, 10 = worst imaginable), that is, moderate to severe range. Prior arrangement to enter or having recently entered treatment at any Boston area outpatient rehabilitation clinic was also required. These and other criteria are given as follows.

*Enrollment Criteria*

*Inclusionary*

- Men and women aged 18–65,
- CNSLBP of ≥6 months duration, not attributed to infection, neoplasm, severe radiculopathy (as indicated by frequent severe pain radiating down a leg), fracture, or inflammatory rheumatic process,
- self-rated bothersomeness of back pain self-rated on average over the preceding 6 months ≥3 on an 11-point ordinal scale (0 = none, 10 = worst imaginable),
- prior arrangement to enter a course of outpatient physical therapy for low back pain at a Boston area rehabilitation clinic,
- English language fluency and mental capacity sufficient to provide informed consent and participate in the study.

*Exclusionary*

- Impaired hearing, speech, vision, and mobility sufficient to interfere with participation in the study,
- current or anticipated receipt of payments from Worker's Compensation or other insurance for disability attributed to low back pain,
- prior treatment with any SI therapy (any variety, including structural massage),
- plans to initiate additional treatment for back pain during the period of the study other than outpatient rehabilitation care, particularly massage or other manual therapies (e.g., chiropractic or osteopathic manipulation),
- exclusions for safety: unresolved musculoskeletal pathology of the lower limbs, current pregnancy, any implanted medical device (e.g., cardiopacemaker, shunts), osteoporosis, any hypercoagulation condition, eczema, skin infection, deep vein thrombosis, burns or other acute trauma including unhealed bone fractures or open wounds, psoriasis, psychiatric illness not well controlled, or current episode of exacerbated major depressive disorder,
- exclusions for anticipated lack of therapeutic response: severe radiculopathic pain, prior discectomy or implantation of rods, screws or plates, or fibromyalgia,
- conditions that might confound therapeutic response or outcomes: medication with coumadin or prednisone, chronic steroid medication, daily use of narcotic analgesics, or alcohol or substance abuse,
- conditions that might confound biomechanical data: current diagnosis of balance problems due to vestibular or other neurological impairments, severe or progressive neurological deficits including neuromotor impairment, estrogen supplementation, tricyclic antidepressants (if not on a regular steady dose at least one month prior to enrollment), or any substance that could impair balance,
- conditions that would confound data on inflammatory biomarkers: type I diabetes, Crohn's disease, lupus, inflammatory bowel disease, ulcerative colitis, any other autoimmune disease, cancer, or body mass index ≥ 40,
- any other major medical condition that has not been stabilized or that would impair the patient's ability to complete study activities.

Candidates were recruited through notices posted at Spaulding outpatient rehabilitation clinics and through a publically accessible online registry of interest in clinical studies [22]. The PI (Eric E. Jacobson) screened candidates and enrolled those that met entry criteria. They then proceeded immediately to baseline data collection. The study paid parking expenses and remunerated participants $50 each time they visited the MAL for screening or data collection.

### 2.3. Randomization, Allocation, and Blinding

The study biostatistician (Roger B. Davis) generated randomization sequences in permutated blocks of 6 stratified by gender and sealed the individual assignments in two series of sequentially numbered opaque envelopes, which were color-coded by gender to allow for stratified allocation. These were stored in a locked metal file cabinet to which the investigators (Eric E. Jacobson, Alec L. Meleger, Paolo Bonato, Roger B. Davis) had access only to respond to emergent, severe AE and to complete IRB required reports. All investigators were initially blind to treatment assignment, but maintenance of blinding proved to be infeasible due to limitations of study staffing. A MAL staff member who had no other role in the study did have access in order to perform randomized allocation, which was done following each participant's baseline data collection. Neither the participants themselves nor the study therapists were blinded due to obvious differences between SI treatment and outpatient physical therapy (OR).

### 2.4. Treatment Protocols

#### 2.4.1. Outpatient Rehabilitation

All participants were required to begin or to continue attending a recently arranged course of outpatient rehabilitation as prescribed by a physician and delivered at any rehabilitation clinic in the Boston area. In general, a typical course of outpatient rehabilitation (OR) for CNSLBP consists of 1/2- to 1-hour sessions twice weekly for 4–6 weeks and may employ various combinations of analgesic and anti-inflammatory medication, joint manipulation, therapeutic exercise, cognitive behavioral treatment, and education. OR treatments were neither administered nor paid for by our study. Because of this we were not able to specify the therapeutic modalities employed, the number or frequency of treatments, or the characteristics of the therapists who delivered them. OR regimens were consequently expected to vary from one clinic to another, but there were no systematic differences between the requirements for OR in the two treatment groups. Participants were allowed 20 weeks to complete their course of OR. The number and frequency of treatments were determined by each participant and their therapist. Compliance with OR treatment was assessed by requesting a list of treatment dates from each participant at 20-week follow-up.

#### 2.4.2. Structural Integration (SI)

SI aims to gradually modify chronic patterns of posture and movement to more closely approximate specific ideals that Rolf put forth as indices of optimum biomechanical efficiency, rather than to focus exclusively on local symptoms. The most important of those ideals are vertical stacking of major body segments (i.e., cranium, thorax, pelvis, and legs), left/right symmetry, horizontality of major body segments on the sagittal plane, and graceful coordination of movement for which thoracic-pelvic counterrotation in gait is often taken as an index. Rolf hypothesized that the individual's ability to alter ingrained patterns of posture and movement was limited by chronic rigidities of the myofascial tissues that envelop all striated muscles. The manipulative technique of SI is believed to reduce the rigidity of these restrictions and, along with increased kinesthetic awareness, to allow the individual to more closely approximate the Rolf ideals [17].

**Figure 1:**
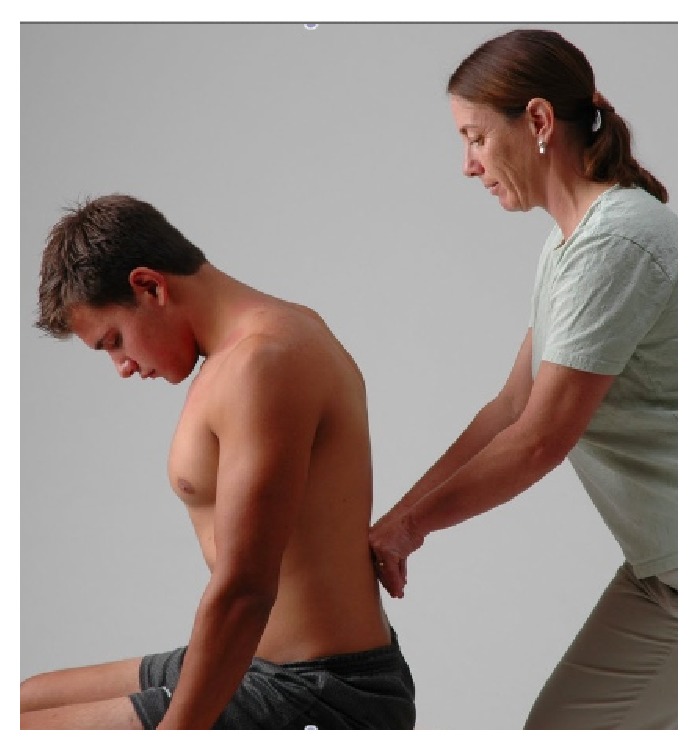
Typical SI manual technique. Courtesy Rolf Institute^*®*^ of Structural Integration.

Most often the client lies on a low, broad treatment table, but is sometimes seated or standing. The therapist uses fingers, knuckles, closed fists, or an elbow to apply sustained pressures and shearing forces of up to several minutes duration to a series of carefully selected, local areas of soft tissue. Often the patient is asked to perform specific movements as force is applied (Figure 1). SI manipulation differs from most forms of massage in the levels of force applied, which may amount to a major fraction of the practitioner's body weight. It differs from chiropractic and osteopathic manipulations in its application of force exclusively to soft tissues. Rolf regarded these features as necessary to induce remodeling of locally restricted myofasciae toward greater elasticity and mobility, but the hypothesis that such remodeling actually occurs has not been scientifically investigated. The SI therapist also teaches awareness exercises that are intended to improve the discrimination of more* versus* less stressful patterns of posture and movement.

As formulated by Rolf, an initial course of SI is delivered in a series of ten sessions known as the Ten Series, which typically includes manipulation of all major joints and anatomical segments. Each of the ten sessions is defined not by specific techniques, but rather by a set of immediate goals for biomechanical change that are intended to advance the approximation of Rolf's more overarching ideals. The aims and anatomical foci of each session are summarized in Table 1. The definition of each session in terms of goals rather than techniques allows the therapist to tailor manipulation and awareness exercises to address individual variations in posture and movement. This individualization of treatment is based on a skilled visual assessment at the beginning of each session. SI was originally provided only by therapists trained at the Rolf Institute^*®*^ of Structural Integration (RISI) [23], but since the mid-1990s a number of other organizations have also provided training at widely varying levels of quality. Adequate training requires at least 300 hours.

In our study SI treatments were provided by five qualified therapists under contract. Each met the criteria of graduation from the adequate training programs of the RISI, the Guild for Structural Integration (GSI) [24], or Kinesis Myofascial Integration (KMI) [25]; a minimum of 10 years clinical practice of SI; and membership in the International Association of Structural Integrators^*®*^ [26]. Two of the therapists were male and three female, ranging 10–29 years in clinical experience. One was a graduate of RISI, one was a graduate of GSI, and three were graduates of KMI. The KMI graduates agreed to provide the Rolf Ten Series instead of the twelve sessions taught by KMI, which include the Ten Series.

Ten sessions conforming to the Rolf Ten Series protocol were provided free of charge to each participant assigned to the SI + OR group. Each session lasted approximately 1 hour, and 20 weeks was allowed to receive all ten. Participants scheduled their own treatments with their choice of one of the five therapists at intervals of their own choosing. Treatments were given individually in the therapists' private practice offices and compliance was monitored from invoices that the therapists submitted to the study administrator.

In order to enhance fidelity of treatment to the Ten Series protocol, a senior SI practitioner (Eric E. Jacobson) led the therapists in a series of group discussions and reviews prior to the beginning of enrollment and also conducted monthly supervision sessions during the treatment phase of the study. However, there was no systematic collection of data on treatment fidelity.

### 2.5. Outcomes

The primary outcome was change between baseline and 20-week follow-up on a patient-rated visual analog scale (0–100 mm) of bothersomeness of pain on average over the preceding week with anchors at “none” = 0 and “worst imaginable” = 100 (VAS Pain) [27]. The secondary outcome was change in the total of the Roland-Morris Disability Questionnaire (RMDQ) over the same period [28, 29]. Exploratory outcomes included the Short Form 36 Health Survey (SF36) [30], the sum of days and half days disabled over the past week, which was calculated from numeric responses to two questions adapted from Deyo et al. [31] (see items 2 and 3 of the Patient's Questionnaire; see the appendix), and Global Satisfaction with Care (GSC), a 7-point Likert scaled response to the question “Over the course of treatment for your low back pain in this study, how would you rate your overall medical care?” the last rated only at week 20 follow-up. SF36 composite scores were obtained using an online calculator, selecting the provided normative reference data “United States, 1998” [32]. These instruments are all patient-completed and have been recommended for use together in low back pain trials [31, 33]. The Wilcoxon rank sum test was prespecified to compare change scores for each of these outcomes across treatment groups.

In addition, four psychological-cognitive variables thought to be prognostic of low back pain chronicity [34] were measured at baseline: the Hospital Anxiety and Depression Scale [35, 36], Pain Catastrophizing Scale [37], Tampa Scale of Kinesiophobia [38, 39], and Wiley-7, a scale for hypochondriasis and somatization [40].

#### 2.5.1. Adverse Event (AE) Monitoring

AE were monitored through reports submitted by study staff and a biweekly Patient Questionnaire (PQ) (see the appendix). The latter was drafted by the PI (Eric E. Jacobson) in response to an IRB requirement that we monitor pain ratings on a biweekly basis, because no prior instrument was available to assess AE associated with SI. This is its first application, and it has not been previously validated. The PQ includes the identical rating questions for VAS Pain and days and half days disabled as the outcome measures administered at baseline and follow-up. It also includes a list of potential treatment related experiences, both positive and negative, each of which may be endorsed as having occurred since the previous questionnaire. For each endorsed experience, an opportunity is provided to rate its duration and to enter a free text description.

AE were inferred from PQ endorsements which were a negative experience, not a symptom of low back pain, and lasting more than one hour. PQ responses were screened against these criteria by two investigators (Eric E. Jacobson, Alec L. Meleger) working independently, with differences resolved by discussion and inspection of the questionnaire responses. All endorsements on each dated PQ return that met the criteria were assumed to refer to the same AE and were attributed to the treatment group to which that participant had been assigned. AE were rated monthly and jointly by the same two investigators (Eric E. Jacobson, Alec L. Meleger) for study-relatedness (definitely, probably, possibly, or not), for seriousness (mild, moderate, or severe), and as expected or unexpected. It was not feasible to blind raters because the IRB required us to report the treatment assignment of each participant with an AE on a monthly basis. The potential for bias in ratings was mitigated by the use of objective criteria for all rating distinctions other than that between mild and moderate seriousness which was made by Alec L. Meleger on the basis of his experience as director of an outpatient rehabilitating clinic.

An IRB mandated stopping rule required us to halt the study if >30% of enrolled participants reported VAS Pain scores ≥30 mm above baseline on two successive PQs. We recorded all such elevations as AE, rated them as study-related and expected, and did not rate them for seriousness.

#### 2.5.2. Other Feasibility Outcomes

We also collected feasibility data on the demographic characteristics of unenrolled* versus* enrolled candidates, compliance with treatment regimens and data collection, and retention to 20-week follow-up (Eric E. Jacobson).

### 2.6. Sample Size

Sample size was estimated using published data from a clinical trial of massage and a meta-analysis of trials of balneotherapy, both for low back pain [41, 42]. A sample of 36 was estimated to provide 50% power to detect a between-group difference in change in VAS Pain of 16.6 mm and 80% power to detect a difference of 23.8 mm, both at the *P* = 0.05 level of significance. Estimates of absolute values for minimal clinically important difference (MCID) for VAS Pain in back pain range from 15 to 19 mm [43–45]. That sample size was also estimated to provide more than 80% power to detect a 5-point difference in change in the RMDQ at the *P* = 0.05 level. MCIDs for RMDQ have been variously estimated as absolute reductions from 2 to 5 points and as a 30% reduction from baseline [43–48]. Allowing for a 10% dropout, a sample of 40 was chosen. This was subsequently increased to 46 in response to an unexpectedly high rate of noncompliance with the requirement to attend OR.

### 2.7. Data Collection and Analysis

All data were collected by the PI (Eric E. Jacobson) at the MAL except for biweekly PQs which were collected by either mail or REDCap, a secure, web-based application designed to support data capture for research studies, that is hosted by Partners HealthCare Research Computing, Enterprise Research Infrastructure & Services group [49]. Missing responses to items on the RMDQ and SF36 were treated according to published recommendations [50, 51]. Because the sample size planned for each treatment group was less than 30, a nonparametric analysis of outcomes was prespecified as a Wilcoxon rank sum comparisons across groups. All analyses were intent-to-treat (ITT) with last observations carried forward. In addition, we performed a* post hoc* ITT responder analysis on VAS Pain data, following recent IMMPACT and Cochrane Back Group guidelines [52, 53]. Given the availability of biweekly VAS Pain ratings which had not been anticipated at the time our analytic plan was registered, we also performed a* post hoc* longitudinal analysis that combined baseline, biweekly PQ, and follow-up data using a linear mixed effects model.

Our prespecified analyses of AE were comparisons across groups of the proportions of participants with at least one study-related AE and the proportions with any AE study-related or not, using Fisher's exact tests. On an* ad hoc* basis we compared the number of study-related AE per participant and separately of any AE per participant using Wilcoxon rank sum. We also compared the proportions with study-related AE rated mild, moderate, and severe. Finally we compared proportions endorsing pain and nonpain types of AE. All data analysis was performed by Eric E. Jacobson with supervision by Roger B. Davis, using SAS 9.3 for Windows.

## 3. Results

### 3.1. Recruitment, Enrollment, and Participant Flow

**Figure 2:**
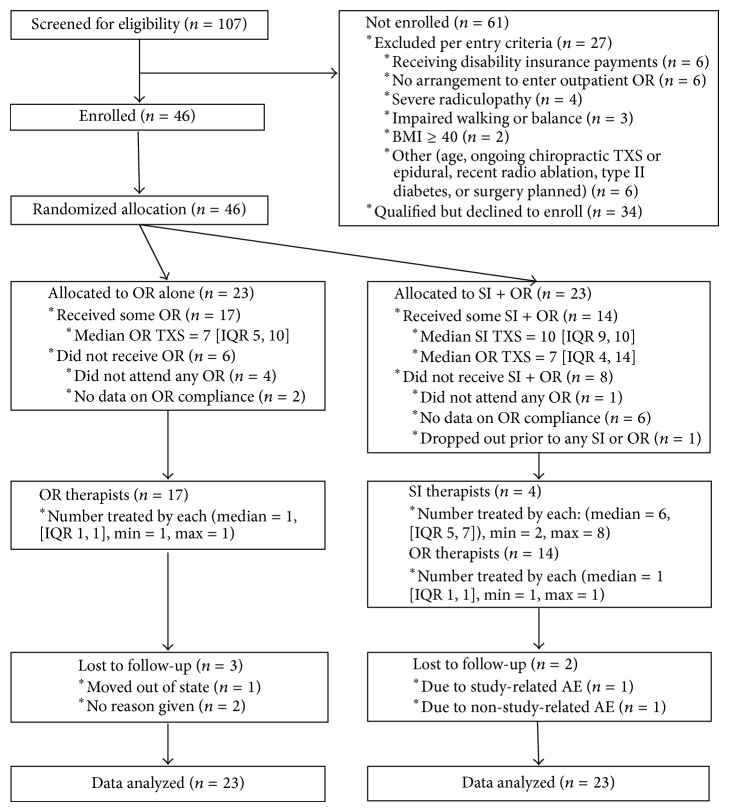
Participant flow. BMI: body mass index; IQR: interquartile range; TXS: treatments.

Screening of candidates began on April 12, 2011, and enrollment was completed on March 8, 2013, for an unexpected total duration of 23 months, which included 4 months during which enrollment activities were suspended. Collection of follow-up data was completed on August 6, 2013. Enrolled* versus* unenrolled candidates were roughly equivalent demographically: enrolled candidates (*n* = 46) were 58.7% female, 78.3% white, and 6.5% Hispanic-Latino and averaged 44.3 years in age. Unenrolled candidates (*n* = 61) were 62.3% female, 81.4% white, and 5.0% Hispanic-Latino and averaged 43.3 years of age. The demographic and prognostic characteristics of the treatment groups were acceptably similar at baseline (Table 2). The flow of participants is displayed in Figure 2.

### 3.2. Compliance and Retention

Compliance with the requirement to receive OR was unexpectedly low but was not significantly different across treatment groups (Table 3). The initial treatment assignment was not altered for any participant, and we found no evidence of crossover.

Loss to 20-week follow-up was 2/23 (9%) in SI + OR and 3/23 (13%) in OR alone, which was not significantly different. (Fisher's exact 2-sided *P* = 1.000) The overall rate of 11% is within the range of 10–20% loss to follow-up that has been recommended as a standard for assessing back pain trials [54].

Three of the 41 participants who provided 20-week follow-up data did so by completing the study questionnaires using the REDCap secure online facility instead of coming to the MAL. Regarding compliance with the biweekly PQ, the difference in numbers of questionnaires returned in SI + OR (median = 7 [IQR 6, 9])* versus* OR alone (7 [4, 8]) was not significantly different. (Wilcoxon rank sum 2-sided *P* = 0.23).

### 3.3. Prespecified Outcomes

**Figure 3:**
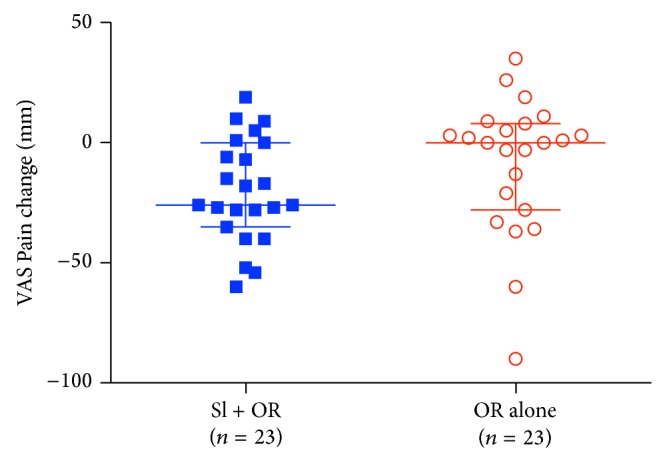
VAS Pain primary outcome. Widest horizontal lines indicate median values; narrower lines indicate interquartile ranges.

**Figure 4:**
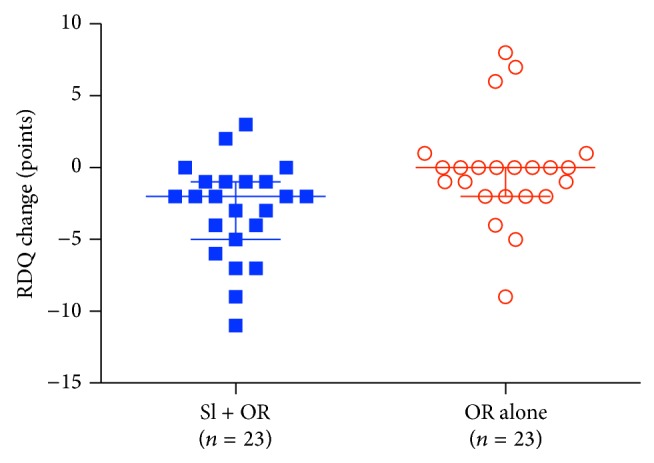
RMDQ secondary outcome. Widest horizontal lines indicate median values; narrower lines indicate interquartile ranges.

The median reductions in VAS Pain, the primary outcome, of −26 mm [IQR − 31.5, −3.0] in SI + OR* versus* 0 mm [−24.5, 6.5] in OR alone were not significantly different (Wilcoxon rank sum 2-sided *P* = 0.075) (Figure 3). However, the difference in median reductions in RMDQ, the secondary outcome, of −2 points [−4.5, − 1] in SI + OR* versus* 0 [−2, 0] in OR alone, were significantly different (*P* = 0.007) (Figure 4). Two points is the smallest suggested absolute MCID for RMDQ [46].

Table 4 summarizes the outcomes. Those with *P* < 0.01 include RMDQ, the SF36 subscale for Bodily Pain, and GSC, each of which had greater improvement in SI + OR. All analyses included each participant in the group to which they were allocated at randomization, *n* = 23 per group.

### 3.4. *Post Hoc* Analyses

In a* post hoc* responder analysis we tabulated the number of participants with reductions relative to baseline that were minimal (10–20%), moderate (≥30%), and substantial (≥50%) and also with absolute reductions of 20 and 40 mm in VAS Pain, using ITT data [52, 53]. We then compared the proportions of responders* versus *nonresponders at each level across groups using Fisher's exact tests. SI + OR had more responders at the minimal and moderate levels at the *P* < 0.05 level of significance. Differences of all other levels were in favor of SI + OR but nonsignificant (Table 5).

**Figure 5:**
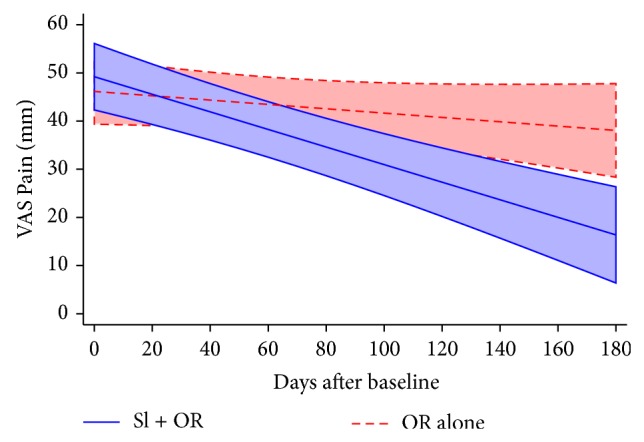
Estimated marginal means and 95% confidence bands for days-group interaction from linear mixed effects model of VAS Pain.

In an additional* post hoc* analysis we constructed a linear mixed effects model of repeated measures data on VAS Pain. A total of 388 observations were available with collection times ranging from 0 to 184 days and an average time from baseline to last observation of 137 days (19.6 weeks). The final model had main effects for baseline VAS Pain, baseline RMDQ, group, days, and the days-group interaction, with random effects for intercept and days. Parameter estimates and their *P* values from that model are given in Table 6. The negative coefficient and small *P* value for the days-group interaction indicate a significantly greater rate of reduction in VAS Pain for SI + OR* versus* OR alone.  Figure 5 displays the estimated marginal means and 95% confidence bands for the days-group interaction.

### 3.5. Adverse Events

A total of 37 study-related AE were attributed to 15/22 (68%) of participants in SI + OR* versus *29 AE to 14/23 (61%) of participants in OR alone. These proportions were not significantly different across groups nor were those for participants with any AE, study-related or not (Table 7). The numbers of AE per participant were also compared across groups using Wilcoxon rank sum, first for study-related AE (*P* = 0.28) and then for all AE (*P* = 0.73), neither being significantly different.

All study-related AE were rated as mild or moderate in seriousness, none were rated as severe, and all were self-limiting; that is, none required medical treatment. The proportions of participants with mild and moderate study-related AE were not significantly different across groups (Table 7). The maximum seriousness of AE for each participant was compared across groups using Wilcoxon rank sum for study-related (*P* = 1.00) and for all AE (*P* = 0.84), neither being significant different.

The most endorsed types of study-related AE in both groups were sharp, burning, and aching pain. The proportions of participants who endorsed the most frequent types were compared across groups, and all were nonsignificant except for a residual category of nonpain endorsements which were significantly more frequent in SI + OR (*P* = 0.005) (Table 7).

No subject reported an elevation of VAS Pain ≥30 mm above baseline on two successive PQ, and the IRB stopping rule was consequently never triggered. Two participants in SI + OR were lost to follow-up due to AE, only one of which was study-related. The first reported an episode of “dread and worry” regarding their next treatment and subsequently dropped out, citing intolerance of the discomfort of SI treatment and a poor relationship with the SI therapist. The second dropped out subsequent to enrollment but prior to receiving any study-related treatment due to an exacerbation of a preexisting medical condition.

## 4. Discussion

This is the first randomized trial to assess the therapeutic effect of SI as an adjunct to OR for CNSLBP and only the third trial of SI for any medical condition [55, 56]. It is the first systematic study of AE associated with SI treatment, which were robustly monitored by both staff and participant reports and identified and rated using conservative criteria.

Regarding feasibility outcomes, we successfully recruited and enrolled a sample whose demographic characteristics did not differ significantly from those unenrolled. Randomization produced treatment groups that were acceptably equivalent on prognostically significant variables. Compliance with SI treatment was high, suggesting that any discomfort associated with it did not dissuade the majority of participants assigned to SI + OR from attending. Neither the incidence nor the seriousness of AE was significantly increased by the addition of SI to OR. Compliance with biweekly and follow-up data collection was acceptable and did not vary significantly between treatment groups. Loss to follow-up was within acceptable limits, and we found no evidence of crossover between treatment regimes. However, the length of time to recruit the cohort was unexpectedly long, and compliance with the requirement to receive OR treatment was unexpectedly low. Both would need to be remediated in a follow-up study.

Improvements in the primary outcome, VAS Pain, were not significantly different between treatment groups. However, improvements in the secondary outcome, RMDQ, were significantly greater in SI + OR versus OR alone, with the difference between median change scores just satisfying the lowest recommended absolute MCID [46]. Among exploratory outcomes the SF36 subscale for Bodily Pain and GSC both improved more in SI + OR* versus* OR alone (Table 4). These outcomes suggest that the addition of SI to OR might provide additional reductions of back pain related disability and satisfaction with care at least in the short term.

In the* post hoc* analysis of longitudinal VAS Pain data the estimated coefficient for the group-days interaction (−0.14 mm/day), which indicates a greater rate of decline in SI + OR versus OR alone, was significant (*P* = 0.0039) (Table 6).

Because this is the only clinical trial of SI for any type of low back pain reported to date, its outcomes can only be compared to studies of other modalities of manipulation for this condition. The most relevant context might be the few other trials that have assessed the effect of manual therapies as adjuncts to outpatient rehabilitation for CNSLBP. One randomized trial for chronic low back pain included a comparison between ongoing usual care* versus* osteopathic manipulative therapy plus usual care. The latter produced significantly greater improvements in a VAS of pain at 1-, 3-, and 6-month follow-ups, but there were no significant differences in the RMDQ change [57]. Another randomized trial compared physician consultation alone* versus* consultation plus muscle energy manipulation and exercise. It found significantly greater mean reduction in VAS Pain in the latter group at both 5 and 12 months [58]. These comparisons are of limited validity because of the significant differences between SI and osteopathic or muscle energy techniques in modes of clinical assessment, treatment goals, and manipulative and educational techniques.

Comparison of our AE data with those from other studies is confounded by variation in the criteria and procedures used to identify AE associated with manual therapies [59]. A systematic review of AE associated with chiropractic as well as other kinds of manual therapies found that in cohort studies about 41% of patients reported minor to moderate AE following a treatment, the majority occurring within one day and resolving within two. The incidence of severe events was extremely small and none were catastrophic. The same review found that in active treatment arms of RCTs 22% of patients reported mild or moderate AE, but this was not significantly different from the rate for sham treatment [60]. However, some studies have found much lower rates. A survey of outpatients receiving ≥3 treatments of osteopathic manipulation found that only 8.6% reported mild to moderate AE after treatment, most frequently pain, soreness, headache, or nausea [61]. Some RCTs of massage for LBP have also reported rates much lower than ours. One noted minor AE such as pain or discomfort in 13% of patients receiving massage [62]. Another comparing relaxation massage, structural massage, and usual care found 4% of relaxation and 7% of structural massage recipients reported AE, mostly increased pain [63]. The higher rates found for both SI + OR (68%) and OR alone (61%) in our study might be due to prompting by the PQ, which invited endorsements of a list of 21 possible dysphoric sequalae to treatment, and to our conservative criterion of >1-hour duration for identifying AE from those endorsements.

### 4.1. Limitations

The large number of exclusion criteria, including those which excluded candidates with systemic inflammatory conditions and neurologically or pharmacologically impaired balance, might have resulted in the enrollment of a sample that was not representative of the typical clinical population, and this might limit the generalizability of our results. Obvious differences between the experiences of OR and SI treatment made it infeasible to blind participants or therapists to treatment allocation. Effective maintenance of the initial blinding of investigators proved to be infeasible due to limited administrative staffing. The potentially biasing effect of the latter was mitigated by the fact that all outcomes were patient-rated. Bias in the identification and rating of AE was mitigated by the use of objective criteria except for the distinction between mild and moderate seriousness. Statistical power to detect absolute MCIDs in the primary and secondary outcomes was limited.

Compliance with the requirement to receive OR was unexpectedly low and might have contributed to the median change scores of zero for both VAS Pain and RMDQ in the OR alone group. For the same reason, intent-to-treat analysis might not provide as good an estimate of the effect of adding SI to OR as would a* per protocol *analysis that used data only from participants who attended some number of OR treatment sessions that had been determined* a priori*. Similar rates of noncompliance with outpatient care for low back pain (~50%) have been reported elsewhere, but our study design also contributed to this problem in that OR was prescribed and delivered outside of our administrative purview [64]. For the same reason we were unable to monitor the specific treatment modalities utilized in OR. Our requests for data on OR attendance from participants often went unanswered. We did not directly monitor SI treatment sessions for fidelity to protocol nor require the therapists to report their treatment interventions in detail.

Because this study assessed the effect of SI as an adjunct to OR* versus *OR alone, its outcomes cannot be interpreted as indicative of the effect that SI alone might have on CNSLBP. The Hawthorne (time and attention) effect was likely greater in SI + OR* versus* OR alone because of the additional 10 hours of hands-on treatment in the former, and this might have contributed to the greater reduction in RMDQ and the slightly higher GSC scores in the SI + OR group. A placebo effect might also have contributed to outcome differentials, because members of the SI + OR group were aware that they were receiving the investigational treatment. Finally, the multiple comparisons made in our analysis of outcomes and the absence of follow-up at a longer duration are additional limitations.

A follow-up study should provide SI according to a specific treatment protocol such as the Rolf Ten Series, should utilize therapists who are adequately trained and experienced in whatever protocol is used, and should allow for the individualization of treatment strategies to reflect actual practice. The collection of information on the specific SI techniques employed in each treatment session would enable closer monitoring of fidelity to protocol. If outpatient rehabilitation were to be a comparator, providing it within the study administration would enable better monitoring of compliance and the collection of data on the specific treatment modalities used. At least a three-month follow-up should be included. We speculate that SI alone might be superior to outpatient rehabilitation alone and note that a direct comparison of the two could control for time, attention, and cutaneous stimulation across treatment groups.

Our positive outcome for greater reduction in disability in the SI + OR group suggests that hypothesized mechanisms for mediating a therapeutic effect of SI are also worthy of future investigation.

## 5. Conclusions

Data on enrollment, retention, data collection, and compliance with SI treatment suggest that a follow-up study would be feasible. However, the study design would have to increase the efficiency of recruitment and improve compliance with OR. The outcomes suggest that adding SI to outpatient rehabilitation for CNSLBP is not likely to enhance reductions in patient-rated pain but is likely to enhance reductions in low back pain related disability at least for the short term and to modestly increase patient satisfaction without significantly increasing the rates or seriousness of AE. If these indications were confirmed by a more definitive study, that might support the recommendation of SI as an effective adjunct to outpatient rehabilitation for CNSLBP.

## Figures and Tables

**Table 1:** Rolf Ten Series treatment goals.

Session	Areas increase pliability, mobility, and L/R and A/P balance
1	(i) Anterior aspect of rib cage and shoulder girdle (ii) Attachments to lateral iliac crests and greater trochanters (iii) Hamstrings, iliotibial bands

2	(i) Feet, ankles, and knees (ii) Anterior aspect of calves and thighs

3	(i) Lateral aspect of the pelvis, torso, and shoulder girdle (ii) Increased independence of thorax from pelvis (iii) Increased range of humerus relative to scapula. (iv) Increased independence of shoulder girdle from neck to rib cage

4	(i) Medial aspect of legs and floor of pelvis

5	(i) Anterior aspect of the pelvis, hips, torso, and lumbar spine

6	(i) Posterior aspect of ankle, leg, knee, hip, pelvis, and lumbar spine

7	(i) Soft tissues spanning the cervical spine and cranium, cranial structure including jaw

8	(i) Promote functional integration between upper extremities, shoulder girdle, and spine

9	(i) Promote functional integration between lower extremities, pelvic girdle, and spine

10	(i) Further optimize functional integration of extremities, shoulder, and pelvic girdles to spine

	Goals for work at end of each of the ten sessions
	(i) Promote physiologic movement of dorsal & lumbar vertebrae
	(ii) Promote physiological movement, L/R and A/P balance of sacrum and 4th and 5th lumbar vertebrae
	(iii) Promote physiologic movement, L/R and A/P balance of cervical spine

L/R: left to right.

A/P: anterior to posterior.

**Table 2:** Baseline characteristics of treatment groups.

Variable (range)	SI + OR (*n* = 23)	OR alone (*n* = 23)	*P* ^1^
Female			
Number (%)	13 (56%)	14 (61%)	0.77
White			
Number (%)	20 (87%)	16 (70%)	0.16
Age years (18–54)			
Mean (SD)	43.1 (13.4)	45.6 (14.0)	0.54
BMI (<40)			
Mean (SD)	26.1 (4.4)	23.0 (4.8)	0.18
VAS Pain (0–100 mm)			
Mean (SD)	46 (23)	50 (20)	0.55
RMDQ (0–24)			
Mean (SD)	7.7 (4.5)	7.7 (5.3)	1.00
Prescription pain medication			
Number (%)	2 (9%)	5 (22%)	0.23
Years since onset			
Mean (SD)	10.7 (10.9)	6.6 (6.3)	0.12
Sum of days and half days disabled			
Mean (SD)	4.1 (4.6)	5.3 (4.5)	0.92
Depression (HADS-D) (0–21)			
Mean (SD)	3.7 (3.9)	3.8 (3.3)	0.98
Anxiety (HADS-A) (0–21)			
Mean (SD)	5.5 (2.8)	5.8 (3.4)	0.71
Pain catastrophizing (PCS) (0–56)			
Mean (SD)	14.3 (11.4)	15.1 (10.6)	0.80
Kinesiophobia (TSK) (−3 to +48)			
Mean (SD)	14.2 (9.7)	15.4 (8.2)	0.67
Hypochondriasis (W7-IW) (0–3)			
Mean (SD)	0.6 (0.6)	0.6 (0.8)	0.71
Somatization (W7-IC) (0–3)			
Mean (SD)	1.1 (1.0)	0.7 (0.8)	0.26

SD: standard deviation.

BMI: body mass index.

VAS Pain: visual analogue scale of pain.

RMDQ: Roland-Morris Disability Questionnaire.

HADS-D and HADS-A: depression and anxiety subscales of the Hospital Anxiety and Depression Scale; PCS: Pain Catastrophizing Scale.

TSK: Tampa Scale of Kinesiophobia.

W7-IW and W7-IC: Illness Worry and Illness Conviction subscales of the Wiley-7.

^1^2-sided Student's *t*-test.

**Table 3:** Compliance with treatment assignments and loss to follow-up.

	SI + OR (*n* = 23)	OR alone (*n* = 23)	*P*
Compliance with OR treatment			
Number of TXS			
Median [IQR]	7 [4, 14]	7 [5, 10]	0.75^1^
PTS w/≥1 treatment			
Number (%)	14 (61%)	17 (74%)	0.53^2^
Compliance with SI treatment			
Number of TXS			
Median [IQR]	10 [9, 10]	0 [0, 0]	
PTS w/≥1 treatment			
Number (%)	22 (96%)	0 (0%)	
Lost to week 20 follow-up	2 (9%)	3 (13%)	1.000^2^

TXS: treatments.

IQR: interquartile range.

PTS: participants.

^1^2-sided Wilcoxon rank sum test; ^2^2-sided Fisher's exact test.

**Table 4:** Outcomes.

	Change scores		
Outcomes (range)	median [IQR]		*P* ^1^
	SI + OR (*n* = 23)	OR alone (*n* = 23)	
Primary outcome			
VAS Pain (0–100 mm)	−26 [−31.5, −3.0]	0 [−24.5, 6.5]	0.075
Secondary outcome			
RMDQ (0–24 points)	−2 [−4.5, −1]	0 [−2, 0]	0.007^4^
Exploratory outcomes			
Days + half days disabled (0–14)	−1.0 [−3.5, 0]	0.0 [4.5, 0.5]	0.445
SF36 subscales (0–100)^2^			
Physical function	5 [0, 15]	5 [0, 13]	0.842
Role physical	25 [0, 50]	0 [0, 25]	0.349
Bodily Pain	16 [7, 25]	0 [0, 11]	0.009^4^
General health	0 [0, 8]	3 [0, 10]	0.673
Vitality	8 [0, 16]	0 [−5, 5]	0.034
Social function	0 [0, 16]	0 [−13, 0]	0.041
Role emotional	0 [0, 0]	0 [0, 0]	0.771
Mental health	0 [−4, 8]	0 [−4, 4]	0.305
SF36 composite scores^2^			
Physical	3 [1, 10]	3 [0, 9]	0.306
Mental	0 [−3, 3]	0 [−4, 1]	0.424
GSC (Likert −3 to +3)^3^	3 [2, 3]	2 [1, 2.25]	0.0003^5^

IQR: interquartile range.

VAS: Visual Analog Scale.

RMDQ: Roland-Morris Disability Scale.

SF36: Short Form (36) Health Survey.

GSC: Global Satisfaction with Care.

^1^Wilcoxon rank sum 2-sided; ^2^higher scores on SF36 subscales and composite scores indicate more positive health; ^3^higher scores on GSC indicate greater satisfaction with care; ^4^ *P* < 0.01; ^5^ *P* < 0.001.

**Table 5:** VAS Pain responders.

	Responders			
Reduction	number (%)		RR (CI)^1^	*P* ^1^
	SI + OR (*n* = 23)	OR alone (*n* = 23)		
10–20%	17 (74%)	9 (39%)	1.89 (1.07–3.32)	0.036^2^
≥30%	15 (65%)	7 (30%)	2.14 (1.08–4.26)	0.038^2^
≥50%	12 (52%)	6 (26%)	2.00 (0.91–4.41)	0.130
≥20 mm	12 (52%)	7 (30%)	1.71 (0.83–3.56)	0.231
≥40 mm	5 (22%)	2 (9%)	2.50 (0.54–11.60)	0.414

RR: relative risk.

CI: 95% confidence intervals.

^1^Fisher's exact 2-sided; ^2^ *P* < 0.05.

**Table 6:** Parameter estimates from linear mixed effects model of longitudinal VAS Pain data.

Parameter	Estimate	SE	*P* ^1^
Intercept	31.53 mm	4.75	n/a
Group^2^	3.06 mm	4.94	0.5364
RMDQ	1.91 mm	0.43	<0.0001^3^
Days	−0.05 mm/day	0.03	<0.0001^3^
Days-group^2^	−0.14 mm/day	0.05	0.0039^4^

SE: standard error.

^1^Wald type 3 F tests of fixed effects; ^2^Group parameters estimate the amount by which the values for SI + OR differ from those for OR alone; ^3^ *P* < 0.001; ^4^ *P* < 0.01.

**Table 7:** Participants with AE.

	Participants			
	number (%)		RR (CI)^2^	*P* ^2^
	SI + OR (*n* = 22)^1^	OR alone (*n* = 23)		
Participants with ≥1 AE				
Study-related AE	15 (68%)^1^	14 (61%)	1.12 (0.73–1.73)	0.76
Any AE	16 (70%)^3^	16 (70%)	1.00 (0.68–1.47)	1.00
Participants endorsing study-related AE by severity				
Mild	12 (55%)^1^	7 (30%)	1.79 (0.87–3.70)	0.14
Moderate	10 (45%)^1^	11 (48%)	0.95 (0.51–1.78)	1.00
Serious	0	0		
Participants endorsing types of study-related AE				
Sharp, burning, aching, or other pain	13 (59%)^1^	9 (39%)	1.51 (0.81–2.80)	0.24
VAS Pain rating ≥30 mm above baseline	4 (18%)^1^	6 (26%)	0.70 (0.23–2.14)	0.72
Numbness	3 (14%)^1^	2 (9%)	1.57 (0.29–8.51)	0.67
Tingling	5 (23%)^1^	2 (9%)	2.6 (0.56–12.10)	0.24
Pulsating sensation	2 (9%)^1^	2 (9%)	1.05 (0.16–6.78)	1.00
Heat, sweating, feeling dizzy or spinning, less coordinated walking, less secure on feet, more difficulty to move, and other nonpain events	12 (55%)^1^	3 (13%)	4.18 (1.36–12.84)	0.005^4^

AE: adverse event; RR: risk ratio; CI: 95% confidence interval; ^1^ *n* = 22 because one participant dropped out before receiving any treatment; ^2^RR, CI, and 2-sided *P* from Fisher's exact tests; ^3^ *n* = 23 to include 1 drop out with a non-study-related AE; ^4^ *P* ≤ 0.01.

## Appendix

### A. Patient's Questionnaire

Please complete the following questions privately so that your answers are not influenced by anyone else.

Thank you!

Please enter the date you completed this questionnaire:

— (yyyy-mm-dd)

#### A.1. How Bothersome Has Your Low Back Pain Been over the Past Week?

Move the slider along the line to the place between “not at all” and “worst imaginable” that shows your answer.

#### A.2. Please Answer the following Questions by Selecting the Answer That Is Right for You from the Drop Down Menus

During the past week, how many days was your activity restricted due to low back problems?
  □ 0
  □ 1
  □ 2
  □ 3
  □ 4
  □ 5
  □ 6
  □ 7
  (select from menu)
During the past week, how many half-days did you spend either in bed, home from work or school, or cutting down on usual activities because of low back pain?
  □ no half days
  □ one half day
  □ two half days
  □ three half days
  □ four half days
  □ five half days
  □ six half days
  □ seven half days
  □ eight half days
  □ nine half days
  □ ten half days
  □ eleven half days
  □ twelve half days
  □ thirteen half days
  □ fourteen half days
  (select from drop down menu)

#### A.3. If You Have Had Any of the Experiences Listed below during or following Your Most Recent Treatment, Please Check the Box Next to It. Only Check Experiences That You Definitely Had

□ (1) Numbness
  Duration during treatment
    □ a few minutes
    □ more than a few minutes
    □ most of the session
  Duration after treatment
    □ a few minutes
    □ more than a few minutes up to an hour
    □ more than one hour
    □ about one day
    □ several days
    □ one week or more
  Please tell us more —
□ (2) Tingling
  Duration during treatment
    □ a few minutes
    □ more than a few minutes
    □ most of the session
  Duration after treatment
    □ a few minutes
    □ more than a few minutes up to an hour
    □ more than one hour
    □ about one day
    □ several days
    □ one week or more
  Please tell us more —
□ (3) Heat
  Duration during treatment
    □ a few minutes
    □ more than a few minutes
    □ most of the session
  Duration after treatment
    □ a few minutes
    □ more than a few minutes up to an hour
    □ more than one hour
    □ about one day
    □ several days
    □ one week or more
  Please tell us more —
□ (4) Cold
  Duration during treatment
    □ a few minutes
    □ more than a few minutes
    □ most of the session
  Duration after treatment
    □ a few minutes
    □ more than a few minutes up to an hour
    □ more than one hour
    □ about one day
    □ several days
    □ one week or more
  Please tell us more —
□ (5) Sweating
  Duration during treatment
    □ a few minutes
    □ more than a few minutes
    □ most of the session
  Duration after treatment
    □ a few minutes
    □ more than a few minutes up to an hour
    □ more than one hour
    □ about one day
    □ several days
    □ one week or more
  Please tell us more —
□ (6) Breathing fast
  Duration during treatment
    □ a few minutes
    □ more than a few minutes
    □ most of the session
  Duration after treatment
    □ a few minutes
    □ more than a few minutes up to an hour
    □ more than one hour
    □ about one day
    □ several days
    □ one week or more
  Please tell us more —
□ (7) Breathing very slow or stopped for a moment
  Duration during treatment
    □ a few minutes
    □ more than a few minutes
    □ most of the session
  Duration after treatment
    □ a few minutes
    □ more than a few minutes up to an hour
    □ more than one hour
    □ about one day
    □ several days
    □ one week or more
  Please tell us more —
□ (8) Feeling dizzy or a spinning sensation
  Duration during treatment
    □ a few minutes
    □ more than a few minutes
    □ most of the session
  Duration after treatment
    □ a few minutes
    □ more than a few minutes up to an hour
    □ more than one hour
    □ about one day
    □ several days
    □ one week or more
  Please tell us more —
□ (9) Feeling an emotion (please write in the name)
  Duration during treatment
    □ a few minutes
    □ more than a few minutes
    □ most of the time
  Duration after treatment
    □ a few minutes
    □ more than a few minutes up to an hour
    □ more than one hour
    □ about one day
    □ several days
    □ one week or more
  Please tell us more —
□ (10) Trembling or shaking
  Duration during treatment
    □ a few minutes
    □ more than a few minutes
    □ most of the session
  Duration after treatment
    □ a few minutes
    □ more than a few minutes up to an hour
    □ more than one hour
    □ about one day
    □ several days
    □ one week or more
  Please tell us more —
□ (11) Remembering something from your past
  Duration during treatment
    □ a few minutes
    □ more than a few minutes
    □ most of the session
  Duration after treatment
    □ a few minutes
    □ more than a few minutes up to an hour
    □ more than one hour
    □ about one day
    □ several days
    □ one week or more
  Please tell us more —
□ (12) Nausea
  Duration during treatment
    □ a few minutes
    □ more than a few minutes
    □ most of the session
  Duration after treatment
    □ a few minutes
    □ more than a few minutes up to an hour
    □ more than one hour
    □ about one day
    □ several days
    □ one week or more
  Please tell us more —
□ (13) Feeling very relaxed
  Duration during treatment
    □ a few minutes
    □ more than a few minutes
    □ most of the session
  Duration after treatment
    □ a few minutes
    □ more than a few minutes up to an hour
    □ more than one hour
    □ about one day
    □ several days
    □ one week or more
  Please tell us more —
□ (14) Feeling that part of your body was pulsating
  Duration during treatment
    □ a few minutes
    □ more than a few minutes
    □ most of the session
  Duration after treatment
    □ a few minutes
    □ more than a few minutes up to an hour
    □ more than one hour
    □ about one day
    □ several days
    □ one week or more
  Please tell us more —
□ (15) Feeling pleasantly warm
  Duration during treatment
    □ a few minutes
    □ more than a few minutes
    □ most of the session
  Duration after treatment
    □ a few minutes
    □ more than a few minutes up to an hour
    □ more than one hour
    □ about one day
    □ several days
    □ one week or more
  Please tell us more —
□ (16) Feeling much lighter or weightless
  Duration during treatment
    □ a few minutes
    □ more than a few minutes
    □ most of the session
  Duration after treatment
    □ a few minutes
    □ more than a few minutes up to an hour
    □ more than one hour
    □ about one day
    □ several days
    □ one week or more
  Please tell us more —
□ (17) Feeling heavier
  Duration during treatment
    □ a few minutes
    □ more than a few minutes
    □ most of the session
  Duration after treatment
    □ a few minutes
    □ more than a few minutes up to an hour
    □ more than one hour
    □ about one day
    □ several days
    □ one week or more
  Please tell us more —
□ (18) Feeling more coordinated as you walk
  Duration during treatment
    □ a few minutes
    □ more than a few minutes
    □ most of the session
  Duration after treatment
    □ a few minutes
    □ more than a few minutes up to an hour
    □ more than one hour
    □ about one day
    □ several days
    □ one week or more
  Please tell us more —
□ (19) Feeling less coordinated as you walk
  Duration during treatment
    □ a few minutes
    □ more than a few minutes
    □ most of the session
  Duration after treatment
    □ a few minutes
    □ more than a few minutes up to an hour
    □ more than one hour
    □ about one day
    □ several days
    □ one week or more
  Please tell us more —
□ (20) Feeling more secure on your feet
  Duration during treatment
    □ a few minutes
    □ more than a few minutes
    □ most of the session
  Duration after treatment
    □ a few minutes
    □ more than a few minutes up to an hour
    □ more than one hour
    □ about one day
    □ several days
    □ one week or more
  Please tell us more —
□ (21) Feeling less secure on your feet
  Duration during treatment
    □ a few minutes
    □ more than a few minutes
    □ most of the session
  Duration after treatment
    □ a few minutes
    □ more than a few minutes up to an hour
    □ more than one hour
    □ about one day
    □ several days
    □ one week or more
  Please tell us more —
□ (22) Easier to breathe
  Duration during treatment
    □ a few minutes
    □ more than a few minutes
    □ most of the session
  Duration after treatment
    □ a few minutes
    □ more than a few minutes up to an hour
    □ more than one hour
    □ about one day
    □ several days
    □ one week or more
  Please tell us more —
□ (23) More difficult to breathe
  Duration during treatment
    □ a few minutes
    □ more than a few minutes
    □ most of the session
  Duration after treatment
    □ a few minutes
    □ more than a few minutes up to an hour
    □ more than one hour
    □ about one day
    □ several days
    □ one week or more
  Please tell us more —
□ (24) Easier to move
  Duration during treatment
    □ a few minutes
    □ more than a few minutes
    □ most of the session
  Duration after treatment
    □ a few minutes
    □ more than a few minutes up to an hour
    □ more than one hour
    □ about one day
    □ several days
    □ one week or more
  Please tell us more —
□ (25) More difficult to move
  Duration during treatment
    □ a few minutes
    □ more than a few minutes
    □ most of the session
  Duration after treatment
    □ a few minutes
    □ more than a few minutes up to an hour
    □ more than one hour
    □ about one day
    □ several days
    □ one week or more
  Please tell us more —
□ (26) Feeling like you were outside your body.
  Duration during treatment
    □ a few minutes
    □ more than a few minutes
    □ most of the session
  Duration after treatment
    □ a few minutes
    □ more than a few minutes up to an hour
    □ more than one hour
    □ about one day
    □ several days
    □ one week or more
  Please tell us more —
□ (27) Feeling like you were more inside your body.
  Duration during treatment
    □ a few minutes
    □ more than a few minutes
    □ most of the session
  Duration after treatment
    □ a few minutes
    □ more than a few minutes up to an hour
    □ more than one hour
    □ about one day
    □ several days
    □ one week or more
  Please tell us more —
□ (28) Sharp pain
  Duration during treatment
    □ a few minutes
    □ more than a few minutes
    □ most of the session
  Duration after treatment
    □ a few minutes
    □ more than a few minutes up to an hour
    □ more than one hour
    □ about one day
    □ several days
    □ one week or more
  Please tell us more —
□ (29) Burning pain
  Duration during treatment
    □ a few minutes at most
    □ more than a few minutes
    □ most of the session
  Duration after treatment
    □ a few minutes
    □ more than a few minutes up to an hour
    □ more than one hour
    □ about one day
    □ several days
    □ one week or more
  Please tell us more —
□ (30) Aching pain
  Duration during the treatment
    □ a few minutes at most
    □ more than a few minutes
    □ most of the session
  Duration after treatment
    □ a few minutes
    □ more than a few minutes up to an hour
    □ more than one hour
    □ about one day
    □ several days
    □ one week or more
  Please tell us more —

#### A.4. Other Experiences

Please describe any other unusual positive or negative experiences in the space below. Also tell us when they started and how long they lasted.
